# Characterization of a Piezoelectric Acoustic Sensor Fabricated for Low-Frequency Applications: A Comparative Study of Three Methods

**DOI:** 10.3390/s23052742

**Published:** 2023-03-02

**Authors:** María Campo-Valera, Rafael Asorey-Cacheda, Ignacio Rodríguez-Rodríguez, Isidro Villó-Pérez

**Affiliations:** 1 Department of Information and Communication Technologies, Universidad Politécnica de Cartagena (UPCT), 30202 Cartagena, Spain; 2 Department of Communications Engineering, Universidad de Málaga, 29010 Málaga, Spain; 3 Department of Electronics and Computer Technology and Projects, Universidad Politécnica de Cartagena (UPCT), 30202 Cartagena, Spain

**Keywords:** ultrasound, piezoelectric ceramics, sensors characterization, acoustic sensitivity, electrical impedance

## Abstract

Piezoelectric transducers are widely used for generating acoustic energy, and choosing the right radiating element is crucial for efficient energy conversion. In recent decades, numerous studies have been conducted to characterize ceramics based on their elastic, dielectric, and electromechanical properties, which have improved our understanding of their vibrational behavior and aided in the manufacturing of piezoelectric transducers for ultrasonic applications. However, most of these studies have focused on the characterization of ceramics and transducers using electrical impedance to obtain resonance and anti-resonance frequencies. Few studies have explored other important quantities such as acoustic sensitivity using the direct comparison method. In this work, we present a comprehensive study that covers the design, manufacturing, and experimental validation of a small-sized, easy-to-assemble piezoelectric acoustic sensor for low-frequency applications, using a soft ceramic PIC255 from PI Ceramic with a diameter of 10 mm and a thickness of 5 mm. We present two methods, analytical and numerical, for sensor design, followed by experimental validation, allowing for a direct comparison of measurements with simulated results. This work provides a useful evaluation and characterization tool for future applications of ultrasonic measurement systems.

## 1. Introduction

Ultrasound measurement systems have gained significant popularity over the last few decades, and their applications span across several industries, including non-destructive testing (NDT) for predictive maintenance and fault detection [1,2,3,4,5,6,7] medical acoustics for diagnosis and ultrasound scans [8,9,10], and communication and monitoring of marine environments [11,12,13,14,15,16]. Piezoelectric transducers are the primary means of generating acoustic energy in most of these systems. As they possess the unique ability to convert electrical energy into mechanical energy and vice versa through the inverse and direct piezoelectric effect, choosing the appropriate ceramic element is crucial for efficient energy conversion. Generally, the ceramic comprises a piezoelectric disk, PZT (lead zirconate titanate), polarized in the thickness direction, with its thickness determining the resonance frequency of the ceramic. Transducers made of this material are widely used for non-destructive testing due to their small size and low cost [17,18]. However, with advancements in technology, researchers are focusing on developing new transduction devices using composite materials, leading to a significant increase in research activities in this field [9,19].

The transducer design comprises the piezoceramic element, a matching layer to enhance the transmission of acoustic energy to the medium, and a protective housing to prevent damage to the active element. Therefore, designing an efficient transducer requires a careful selection of each of these components [20,21,22]. Ensuring compatibility between these components is particularly important to enhance the frequency bandwidth of the transducer, and even though the manufacturing process of a transducer may appear straightforward, a lack of attention to these design details can quickly make it complex.

Recent advances in piezoelectric transducer technology have led to the manufacturing of transducers in different geometries, including plate type, rod type, ring type, and cylinder type [17,23,24,25], and with varying vibration modes such as transverse, tangential, radial, and others [26,27,28,29,30]. Analytical models have been developed to study the resonance frequencies of these modes in ceramics with different geometries, based on their elastic, dielectric, and electromechanical properties [28,31,32]. Some studies have also employed numerical methods, such as the finite element method (FEM), to obtain accurate modes [33], while others have combined analytical and numerical methods to optimize the vibrations of the ceramic and validate the design [18,34,35]. Recent works [35,36] have studied the vibration characteristics of transducers using disks of different thicknesses to obtain the electrical impedance and natural frequency of the ceramic. Other studies, such as those in [16,23,37], have used optical systems to measure the resonance frequencies of ceramics with free boundary conditions and have compared their results with experimental laboratory measurements.

Although several recent studies have explored the dynamics of piezoelectric transducers, a direct comparison method for characterizing a ceramic/transducer based on its acoustic sensitivity is not currently available in the literature. The only analytical approach described thus far is the use of reciprocity and the pulse-echo method, as discussed in [38]. This characteristic is vital in determining the radiated acoustic power and the received electrical power and is therefore essential to the design and manufacture of effective ultrasonic measurement systems. In this paper, we propose a complete study that utilizes an easy-to-manufacture/assemble piezoelectric acoustic sensor based on the known electrical impedance [34,39] and acoustic sensitivity of a soft ceramic PIC 255 vibrating at low-frequencies. We compare the results using analytical, numerical, and experimental methods to analyze the transient signal received by the acoustic sensor in the low-frequency range of up to ∼170 kHz. This study is intended to serve as a useful evaluation and characterization tool for future ultrasonic measurement system applications.

The paper is organized as follows: In Section 2, we introduce the architecture of the transducer and discuss the analytical and numerical methods used to design the sensor, as well as the fabrication process of the final design. In Section 3, we discuss the characterization methods of the electrical impedance and receiving voltage response (RVR) for both the free ceramic and the sensor, as measured in water. Section 4 presents the experimental results obtained and compares them with the theoretical results. Finally, in Section 5, we offer concluding remarks.

## 2. Design of a Piezoelectric Acoustic Sensor

Traditionally, the design of a piezoelectric ultrasonic transducer is performed following a methodology based on experimental knowledge combined with a theoretical understanding. After an initial design, the configuration of the transducer or its parts is analyzed using simple 1D analytical models or more powerful numerical simulation tools, such as COMSOL Multiphysics, which allows 3D simulations.

Since it is desired to fabricate an ultrasonic transducer for low-frequency applications for transient signals (broadband), which is easy to manufacture, assemble, and small in size, the design aspects (active element, matching layer, and housing) to be taken into account to meet these criteria will be addressed throughout this section.

The main components of a sensor are [15,18]:

1. Active element (ceramic): This is a piezoelectric material with a given geometry depending on the required use, and very thin electrodes in the whole area perpendicular to the surface that receives the acoustic wave to be recorded.
2. Matching layer (ML): They can be one or more layers bonded to the front face of the active element in order to optimize the transmission of acoustic energy between the load and the ceramic, i.e., to adapt the acoustic impedances between the two through the different intermediate layers.
3. Housing: This is the component that closes the whole sensor assembly. To avoid electrical ground differential effects as well as the influence of possible electromagnetic waves, it is usually designed with an electrically conductive material.

### 2.1. Importance of the Ceramic

Piezoelectric ceramics, specifically lead zirconate titanate (PZT), are currently the most widely used in the market due to their low-cost and high electromechanical coupling coefficient and dielectric constant. These ceramics can be classified into two types: soft and hard ceramics. Soft ceramics, known for their ferroelectric behavior and high mobility [40], are often used in piezoelectric sensors due to their ease of polarization and high coupling factors. They find applications in vibration sensors, ultrasonic flow transmitters and receivers, as well as micro- and nanopositioning and electroacoustics [41,42]. On the other hand, hard ceramics can withstand high levels of electrical excitation and are typically used in high-power transducers.

After careful consideration, a soft PZT piezoceramic PIC255 from PI ceramic [43] was chosen as the ideal candidate for low-frequency applications due to its excellent acoustic sensitivity in the desired frequency range (up to ∼170 kHz), and its small size, with a diameter of ∼10 mm and a thickness of ∼5 mm. In designing and applying piezoelectric transducers, the vibration characteristics of the ceramics are important, and it should be noted that piezoelectric disks have three different oscillation modes: transverse, tangential, and radial extensional. However, as described in [37], only the resonance frequencies of the radial extensional modes can be measured in an impedance analysis. Therefore, this paper focuses solely on the analysis of the radial extensional mode (low-frequency), which is sufficient for the proposed application as demonstrated below.

As the piezoelectric transducers are usually circular, the vibration characteristics of the ceramics are important in the design and application of transducers. It is important to highlight that there are three different oscillation modes of piezoelectric disks: transverse, tangential, and radial extensional. However, a theoretical and experimental analysis of these modes, described in [37], shows that only the resonance frequencies of the radial extensional modes can be measured in an impedance analysis. Consequently, in this paper, we only analyze the radial extensional mode (low frequency). This mode is enough for the proposed application as is demonstrated below.

### 2.2. Analytical Method: Design for Circular Piezoelectric Ceramics

It is common practice to analyze the vibration characteristics of piezoelectric ceramics using 1D (one-dimensional) analytical models as they help to give an approximation of the response of a transducer, are computationally very inexpensive and help to speed up product design cycles [23,28,33,44]. According to this, the vibrational behavior of a circular ceramic can be obtained from the constitutive equations.

**Figure 1:**
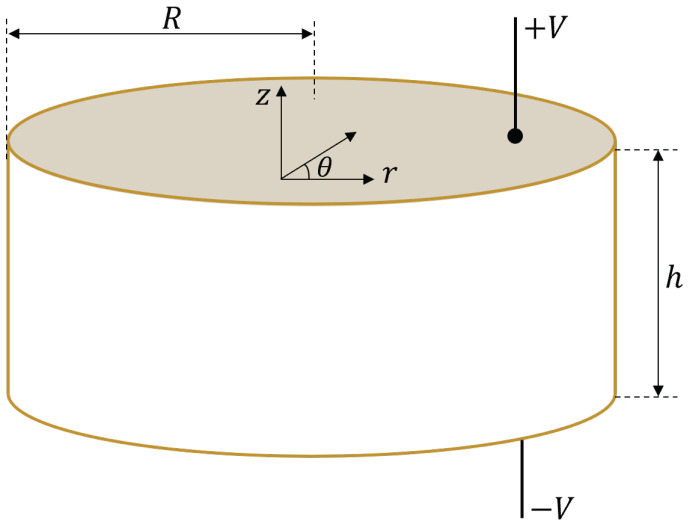
Piezoelectric ceramic with axial polarization, thickness *h*, and radio *R*. The coordinates (r,θ,z) are cylindrical coordinates with the origin in the center of the ceramic.

Figure 1 shows the geometrical scheme of the piezoelectric ceramic of thickness *h* and radius *R*. The ceramic is polarized along the thickness (*Z*-axis), and the two opposite planar faces are covered with full electrodes.

In the following, the cylindrical coordinates (r,θ,z) will be used. If the radial extensional oscillation is assumed to be axisymmetric and harmonic in time, *t*, with a known angular frequency ω, the displacement of the middle plane along the radius *R* of the ceramic, $u_{r}\left(r,t\right)$, can be expressed as a function of the radial component, U(r), as $u_{r}\left(r,t\right)=U\left(r\right)\;e^{j\;\omega \;t}$. In this case, the dynamic behavior is given by the common equation for radial mode analysis in two dimensions [28] whose general solution is:

(1) $$U\left(r\right)=C\;J_{1}\left(\beta \;r\right),$$

where $J_{\alpha }\left(\beta \;r\right)$ is the Bessel function of the first kind and order α, and the parameters *C* and β are defined as:

(2) $$C=\frac{2\;V\;d_{31}\left(1+v_{p}\right)}{\left(1-v_{p}\right)J_{1}\left(\beta \;R\right)-\beta \;R\;J_{0}\left(\beta \;R\right)}\;\cdot \;\frac{R}{h},$$

(3) $$\beta =\omega \sqrt{\rho \;s_{11}^{E}\left(1-v_{p}^{2}\right)},$$

where $v_{p}=s_{12}^{E}/s_{11}^{E}$ is the planar Poisson’s ratio, ρ is the density of ceramic material, *V* is the electrical potential, and $d_{31}$, $s_{12}^{E}$ and $s_{11}^{E}$ are the mechanical and piezoelectric coefficients of the ceramic PIC255 used in this work (Appendix A).

Applying a potential difference *V* between the ceramic electrodes, the electric current *I* for radial extensional oscillation is expressed as [37]:

(4) $$I=j\;\omega \frac{2\;\pi \;R^{2}\;V\;\varepsilon_{33}^{T}}{h}\;\cdot \;\frac{\left[1-v_{p}+\left(1+v_{p}\right)\frac{k_{p}^{2}}{k_{p}^{2}-1}\right]J_{1}\left(\beta \;R\right)-\beta \;R\;J_{0}\left(\beta \;R\right)}{\left(1-v_{p}\right)J_{1}\left(\beta \;R\right)-\beta \;R\;J_{0}\left(\beta \;R\right)}$$

Resonance and antiresonance frequencies in radial oscillation are important characteristics of a piezoelectric ceramic. When a resonance frequency is applied, intensity approaches infinity. On the other hand, an antiresonance frequency makes intensity disappear in the piezoelectric element. Thus, resonance and antiresonance frequencies can be derived from Expression (4), respectively, as follows:

(5) $$\beta \;R\;J_{0}\left(\beta \;R\right)=\left(1-v_{p}\right)J_{1}\left(\beta \;R\right),$$

(6) $$\beta \;R\;J_{0}\left(\beta \;R\right)=\left[1-v_{p}+\left(1+v_{p}\right)\frac{k_{p}^{2}}{k_{p}^{2}-1}\right]J_{1}\left(\beta \;R\right),$$

Thus, this analytical method serves as a basis for comparing the results obtained with both the numeral method and electrical impedance experiments, considering that the impedance reaches a local minimum when the ceramic oscillates at the resonance frequency and a local maximum at the antiresonance frequency.

The particularities of the numerical model are presented below.

### 2.3. Numerical Method: Design for Circular Piezoelectric Ceramics

The finite element method (FEM) is a numerical technique used to find approximate solutions for the equations governing the piezoelectric effect and acoustic wave propagation, involving partial differential equations. Commercial programs, such as COMSOL Multiphysics [45], are commonly used for this purpose, which allows obtaining the vibrational behavior of ceramic in a more realistic way. However, it is crucial to have a strong understanding of the relevant physics, proper mesh resolution to solve the waves, and appropriate boundary conditions to accurately model piezoelectric ceramics.

In this section, we will discuss key considerations for modeling latent piezoelectric ceramics using the FEM.

**Figure 2:**
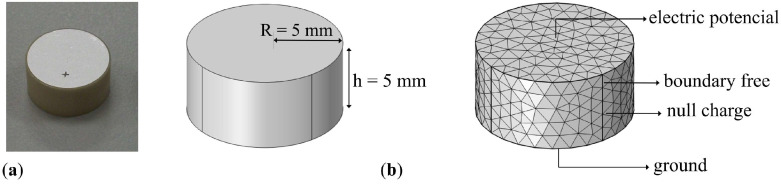
(**a**) PIC 255 ceramic; (**b**) Geometry and 3D finite element meshing of piezoelectric ceramic in COMSOL Multiphysics with radio R=5 mm and thickness h=5 mm.

The preprocessing, processing, and post-processing used in the simulation of the piezoelectric ceramic to obtain the impedance electric and RVR was implemented as follows:

1. Preprocessing: The simulations are performed in the structural mechanics module in combination with the piezoelectric devices interface.
  - Geometry: Geometries with a ratio R≥h can be approximated by a disk. The piezoelectric ceramic cylinder type PIC255 is simulated and dimensioned in 3D. It has a radius R=5 mm, and a thickness of h=5 mm polarized in the longitudinal axis.
  - Frequencies of interest and meshing: In the sizing of the tetrahedral mesh elements, it was taken into account that the minor wavelength (maximum frequency, 250 kHz) was discretized in 16 parts. Thus, the number of mesh elements with tetrahedral structure was 4318. Figure 2 shows the mesh used to discretize the solutions.
  - Boundary conditions:
    – Free: This is the mechanical boundary condition, which applies to all ceramic domain boundaries when the ceramic is free-form.
    – Null charge: Default electrostatic boundary condition, which has no electrical charge on the boundary and therefore applies to the non-electrode side surface of the ceramics.
    – Initial values: These introduce an initial shift of the acoustic field, electric potential, or their derivatives. All initial values are set to 0 and apply to the entire geometry.
    – Axial symmetry: This is a default boundary condition used to obtain such symmetry. It is set on the longitudinal axis of the ceramic.
    – Electric potential: Sets the electric potential to a value of 1 V at one of the electrodes.
    – Ground: Sets the electric potential to zero at the boundary applied to the other electrode surface.
2. Processing: The input parameters are the coefficients of the elasticity matrix, the coupling matrix, the permittivity matrix, the density, and the mechanical and dielectric losses, respectively. For the development of this numerical model the Frequency Domain study is used, where the displacement field and the electric potential can be obtained from $u\left(r,t\right)=u_{t}\left(r\right)e^{j\;\omega \;t}$ and $V\left(r,t\right)=V_{t}\left(r\right)e^{j\;\omega \;t}$.
3. Post-processing: Two quantities are used to characterize the sensor: electrical impedance and RVR.
  - Electrical impedance: The impedance, *Z*, is obtained from the inward surface charge density at one of the electrodes, $\sigma_{n}$, and the potential difference. The electrical impedance can be obtained as follows [39]:
    (7) $$Z=\frac{V}{I}=\frac{V}{\int_{S}\sigma_{n}\;dS},$$
    where *I* is the current intensity across the electrode, being the integral of the inward surface density along the entire surface, *S*, of the electrode.
    Deriving the admittance from the impedance is straightforward (Expression (7)). Its calculation allows us to compare the behavior of the ceramic at the resonance frequency with that of the experimental results.
  - Receiving Voltage Response: In a linear regime, a ceramic radiates an acoustic wave with an amplitude proportional to its emission sensitivity. Moreover, during the acquisition of acoustic waves, it generates an electrical signal proportional to its reception sensitivity.
    During transmission, the ceramic voltage sensitivity, $S_{Tx,v}$, is used to express the pressure *P*, in Pascals, generated in the medium at a distance of 1 m in free field conditions as a function of the input voltage. Thus, given an input voltage, $V_{in}$, $S_{Tx,v}=P/V_{in}$. This parameter is usually expressed in dB, taking as a reference sensitivity 1 μPa/V.
    The relationship between the voltage and intensity sensitivities is defined as $S_{Tx,i}=S_{Tx,v}\;\cdot \;\left|Z_{T}\right|$, where $Z_{T}$ is the electrical input impedance of the ceramic.
    During the reception of acoustic signals, the relationship between the voltage generated in the ceramic when its terminals are in open circuit, $V_{out}$, and the reception of an incident acoustic pressure *P* in Pascals, in a free field, is defined as $S_{Rx}=V_{out}/P$. This parameter is usually expressed in dB, taking as a reference sensitivity 1 μPa/V.
    The reciprocity principle, $R_{cp}$ (denoted in this paper as $R_{cp}$ instead of *J* to avoid confusion), is defined as the relation between the ceramic reception and transmission intensity sensitivities [46]. Moreover, the following must hold:
    (8) $$R_{cp}=\frac{S_{Rx}}{S_{Tx,i}}=-j\frac{2\;\lambda \;x}{\rho \;c},$$
    where λ=c/f is the wavelength, *x* is a reference distance of 1 m, ρ the water density, 1000 kg/$m^{3}$, and *c* is the sound’s propagation velocity in water 1480 m/s.
    From previous expressions, when the type of waves radiated by the transducer and the sensitivity in one of the two directions are known, the sensitivity in the other direction can be derived from the reciprocity principle. For spherical waves, the relationship between the two sensitivities is given by [46] as:
    (9) $$20\log\frac{S_{Rx}}{1\;V/\mu \mathrm{Pa}}=20\log\frac{S_{Tx,i}}{1\;\mu \mathrm{Pa}/V}-354-20\log f\left(k\mathrm{Hz}\right)$$
    From expression (9), it is straightforward to obtain the RVR using the transmission sensitivity of each of the simulated frequency steps. Thus, the numerical model consists of exciting a point sufficiently far away from the ceramic from the calculation of the sensitivity in emission by applying the reciprocity principle.

#### Analysis and Numerical Results

**Figure 3:**
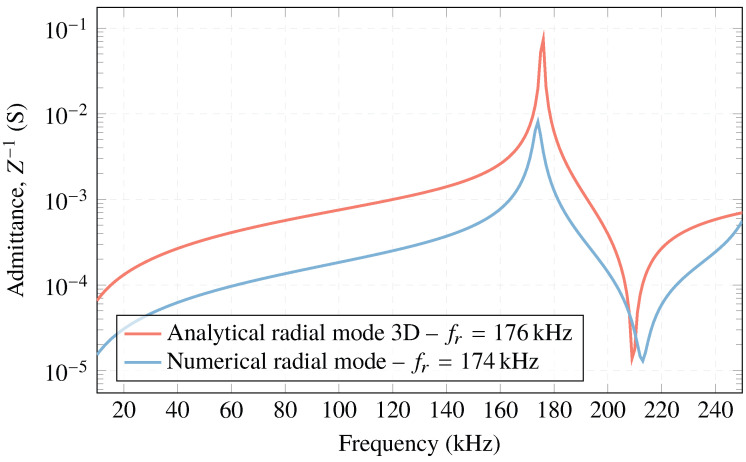
Electrical admittance of the analytical and numerical models for PIC 255 ceramic, R=5 mm and h=5 mm.

Figure 3 compares the results obtained with both the analytical and numerical models. As can be seen, the resonance frequency obtained in the radial oscillation mode with the analytical model, proposed by [37], is $f_{r}=176\;k\mathrm{Hz}$, and the one provided by COMSOL Multiphysics is $f_{r}=174\;k\mathrm{Hz}$ with an error of ±1 with respect to the analytical method. From these results, it can be concluded that both models provide similar resonance frequencies, validating each other.

**Figure 4:**
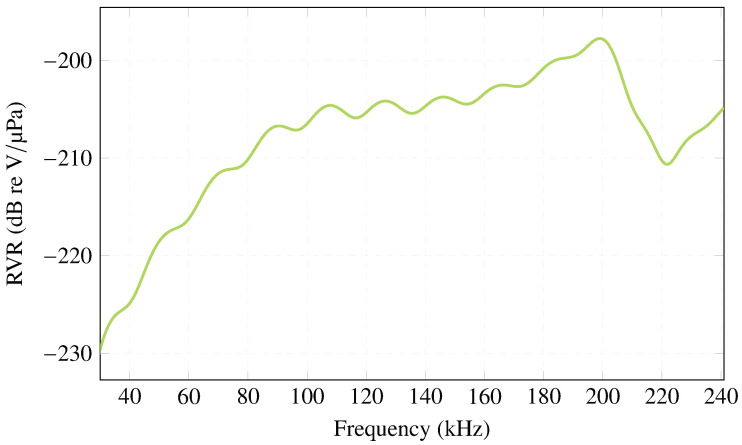
RVR simulated in COMSOL Multiphysics for PIC 255 ceramic, R=5 mm and h=5 mm.

Regarding the RVR, Figure 4 obtained with COMSOL Multiphysics, shows an approximation to the experimental measurements. The plot presents an almost homogeneous behavior in amplitude, with ∼−205 dB re V/μPa starting at 90 kHz.

The similarities of both analytical and simulated models allow us to move closer to the design and manufacturing of the sensor.

### 2.4. Importance of the Matching Layer

The acoustic matching layer is used to increase the efficiency in coupling acoustic energy from the incident medium (water) to the transmission medium (ceramic). It is commonly built with a material that has an acoustic impedance between the incident one of the water, $Z_{i(water)}$, and the transmission one of the ceramic, $Z_{t(ceramic)}$ [21].

In most cases, piezoelectric ceramics have a higher impedance in relation to the acoustic loads (water, tissue, etc.). Thus, much of the ultrasonic energy is reflected back to the load/ceramic interface. This is why acoustic impedance matching layers are used, to achieve better results in the bandwidth response of the designed sensor. To this end, a layer is added between the receiving face of the ceramic and the acoustic load, allowing to increase the mechanical load of the interface.

#### Analysis

1. Zero-layer model: Considering a simple model in which the transmission of an acoustic wave that is generated in a medium and is received by the ceramic is studied (where the electrical signal is recorded), the expected signal loss can be estimated if only the free ceramics are in the water.
  The sound intensity transmission coefficient, $T_{i}$, is derived from the following known expression [47]:
  (10) $$T_{i}=\frac{4Z_{t}/\;Z_{i}}{{\left(Z_{t}/\;Z_{i}+1\right)}^{2}}$$
  In this case, the acoustic wave is generated in the water with $Z_{i(water)}=1.48\;\mathrm{MRayl}$ and received by the ceramic with $Z_{t(ceramic)}=31.2\;\mathrm{MRayl}$, as depicted in Table 1. From Expression (10), i.e., when there is no matching layer, $T_{i}=0.17$. This means only 17% of the signal generated in the medium is finally transmitted to the ceramic.
2. One-layer model: Understanding the importance of using a matching layer to maximize the acoustic transmission between the water and the ceramic, it is necessary to use an intermediate layer that makes the impedance matching progressive.
  For the case $Z_{i}<Z_{1}<Z_{t}$, the best impedance optimizing the transmission is $Z_{1}=\sqrt{Z_{i}\;Z_{t}}$ [18]. Thus, $Z_{1}=6.8\;\mathrm{MRayl}$.

Although it was not the best option, we chose methacrylate as the matching layer with two different thicknesses (5 mm and 10 mm) because it presents an increase in acoustic sensitivity at certain frequencies, as will be explained below.

Regarding methacrylate, it has an acoustic impedance of 3.21 MRayl. This means that optimal transmission cannot be achieved. However, the resulting transmission curves are calculated by attaching the sensors to the methacrylate layer and predicting the increasing sensitivity of the frequencies.

**Figure 5:**
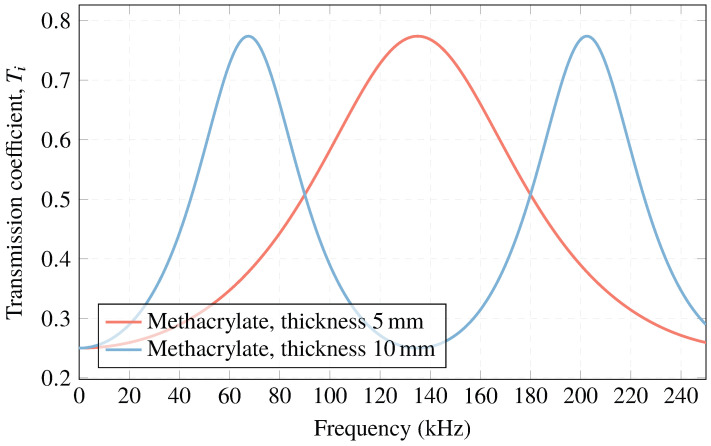
Acoustic transmission coefficient with a methacrylate matching layer.

Figure 5 shows the transmission curves obtained for a matching layer thickness of 5 mm and 10 mm. It can be observed two transmission maxima at 67 kHz and 202 kHz for a thickness of 10 mm of methacrylate, and another maximum of 134 kHz for the thickness of 5 mm. This optimizes the sensor behavior for low frequencies and can provide a transmission coefficient Ti=∼70%.

### 2.5. Importance of the Housing

**Figure 6:**
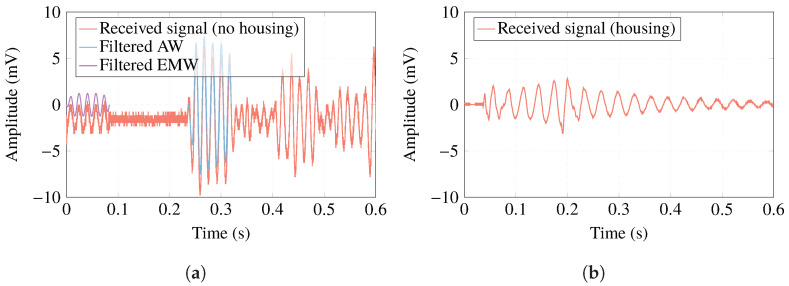
(**a**) Signal received in the process of emission-reception in the piezoelectric ceramic free in water (electromagnetic wave (EMW) in red, acoustic wave (AW) in blue); (**b**) example of the acoustic signal received by housing ceramic.

During acoustic calibration of piezoelectric ceramics measured free in the water, both acoustic and electromagnetic waves can be observed as a result of transmission or reception processes. Figure 6a shows an example of one of the calibration signals. In it, the acoustic wave appears after the reception of the electromagnetic wave. Figure 6b shows an example of what can be observed when the ceramic is covered with housing. The electromagnetic wave disappears, since the housing functions as a Faraday chamber [48] without the Hall effect and the amplitude of this received wave is reduced.

In addition, the housing is used for ease of use of the transducer and mechanical protection of the transducer’s design elements, such as the piezoelectric ceramic and the electrical connections [20].

The design of the housing is carried out using AutoCAD software as an initial approximation, which is then machined using a milling machine to create a physical model. The housing consists of two parts, namely the back and front parts. The back part of the housing serves as a support for the piezoelectric ceramic while the front part is responsible for screwing and safeguarding the interior of the housing against potential leaks. For the manufacturing of the housing, aluminum is chosen as the material of construction due to its various desirable properties, including good thermal and electrical conductivity, light weight, high-temperature resistance, mechanical strength, and corrosion resistance. The use of aluminum also provides a cost-effective solution for housing construction.

### 2.6. Sensor Manufacturing

**Figure 7:**
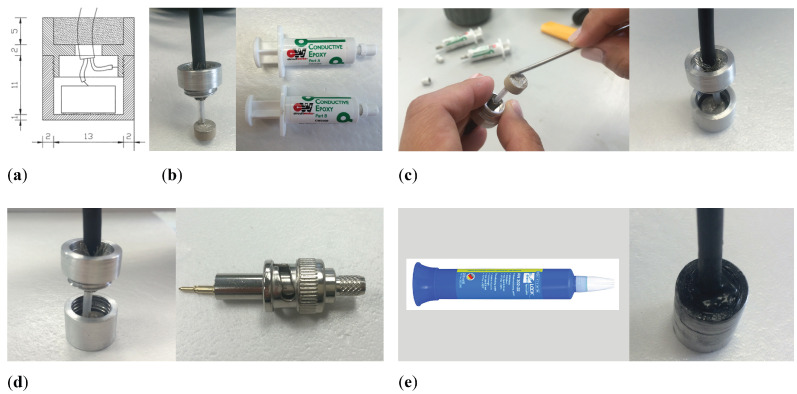
Sensor manufacturing process. (**a**) Design of the housing, measures in millimeters; (**b**) Coaxial cable attached to the positive pole of the ceramics; (**c**) Ceramic bonding to the housing; (**d**) Shielding of the cable with housing; (**e**) Sealed sensor.

Once the aluminum housing is designed as depicted in Figure 7a, the sensor is assembled as illustrated in Figure 7. The first step is to pass a coaxial cable through the hole that closes the transducer and glue what will be the positive pole to the center of one of the faces of the ceramic with conductive epoxy. Then, it is left to dry completely for 24 h as seen in Figure 7b. The other side of the ceramic is glued to the back part of the housing with conductive epoxy, and again left to dry for 24 h, as shown in Figure 7c. The shield of the cable will make contact with the housing. A BNC connector will be used at the end of the cable, (Figure 7d). Finally, the sensor is screwed on and the outer joints are sealed with Weicon Lock Pen Sistem AN 302-43 insulating glue to prevent leakage. The hole that closes the sensor is sealed with Sikaflex, which is a permanently elastic polyurethane-based adhesive material with high water resistance (Figure 7e) and again left to dry for 24 h. Subsequently, experimental measurements in water are carried out with this sensor.

## 3. Experimental Methods Characterization: Ceramic and Sensor

The measurements were conducted using equipment provided by the Escuela Politécnica Superior de Gandía at the Universitat Politècnica de València. Appendix B presents a detailed list of the devices used in the measurements.

**Figure 8:**
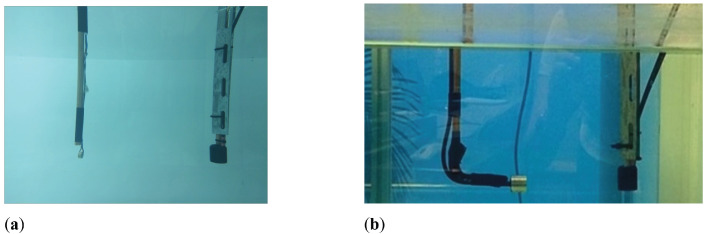
Experimental measurements. (**a**) Free ceramic in water; (**b**) Sensor in water.

Electrical admittance and RVR are fundamental features that define piezoelectric sensors. This section describes the processes for the characterization of this sensor, and the experimental setups consisting of measurements with free ceramic and sensor in water, as shown in Figure 8.

### 3.1. Electrical Admittance

**Figure 9:**
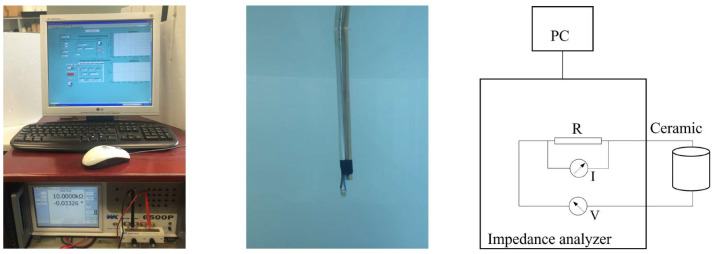
Experimental setup and schematic diagram for impedance measurement.

To measure the electrical admittance, we use the Wayne Kerr Electronics WK6500P high-frequency impedance analyzer Signals are transmitted using a frequency sweep from 10 to 250 kHz in 0.1 kHz steps and a homogeneous amplitude voltage of 500 mV. The response provided by the transducer is characterized by measuring the potential difference. The experimental setup and scheme are shown in Figure 9.

### 3.2. Receiving Voltage Response

**Figure 10:**
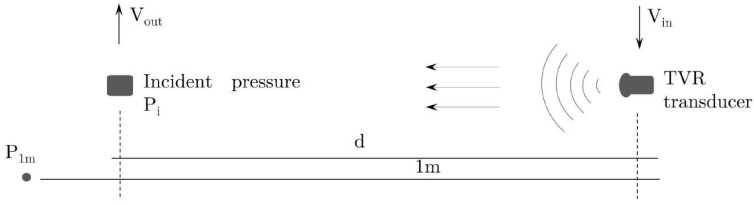
Schematic of the RVR measurement setup.

To obtain the RVR, the direct comparison method was used, with which the receiving response of the designed sensor is obtained based on the known emission response (as discussed in Section 2.3) of a reference transducer. The RVR measurement scheme is shown in Figure 10.

**Figure 11:**
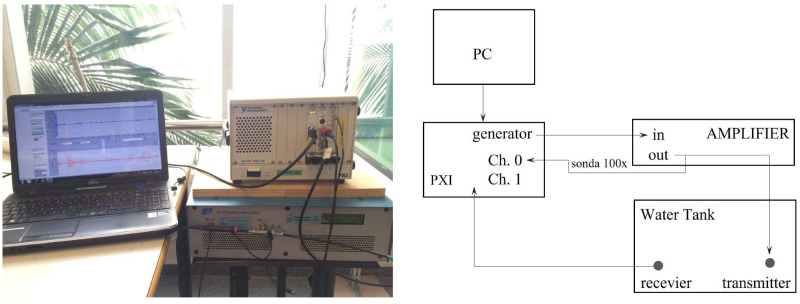
Experimental setup and schematic diagram for measuring the RVR of the piezoelectric sensor.

The measurements were configured using a National Instruments PXI 1031-DC generation and acquisition system, connected to a computer. The signal generates tones ranging from 10 to 250 kHz in steps of 0.1 kHz, is amplified by an E&I 2100L RF, and sent to the transducer SX60-FR with a Transmission Voltage Response (TVR) of 134 dB re Pa/V @ 1 m, located in a water tank with dimensions of 1.20×0.80×0.60$m^{3}$ and at a distance of 23 cm from the sensor. On the other hand, the propagated signals are received by the sensor, converting them into electrical voltage, as depicted in Figure 11.

## 4. Results and Discussion

This section compares the experiments with the analytical and numerical studies for the free ceramic, the in-water sensor of electrical admittance and RVR, as well as the sensor attached to the matching layer.

**Figure 12:**
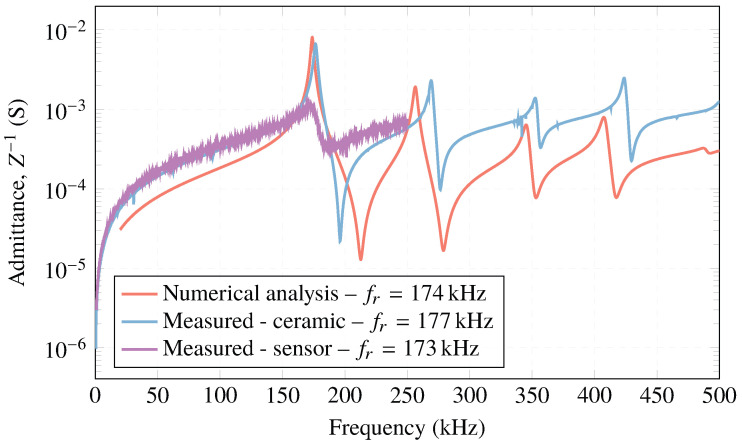
Simulated and measured electrical admittance obtained for the ceramic and sensor in water.

**Figure 13:**
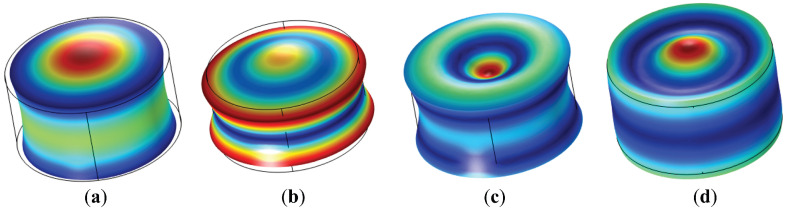
Radial extensional oscillation for PIC ceramic 255. (**a**) first mode 177 kHz; (**b**) second mode 270 kHz; (**c**) third mode 353 kHz; and (**d**) fourth mode 425 kHz.

Regarding the electrical admittance, Figure 12 demonstrates that the ceramic’s resonance frequency, $f_{r}=177\;k\mathrm{Hz}$, is close to the one provided by the numerical simulation, $f_{r}=174\;k\mathrm{Hz}$. For the sensor, there is a decrease of 4 kHz in the resonance frequency, $f_{r}=173\;k\mathrm{Hz}$, compared to the free ceramic measured in water. This is due to the increase in mass and thickness caused by the housing. In addition, the measurement of the free ceramic shows the first four vibration modes at low frequency (radial mode) in 177, 270, 353, and 425 kHz. The different radial modes of oscillation are observed in Figure 13, calculated from the simulations with the commercial software COMSOL Multiphysics.

The first mode in the ceramic presents a slight maximum radial deformation in the central axis and, in turn, another deformation in the longitudinal axis with an elongation of the ceramic (thickness). In the next three modes, the deformations in the radial plane are more evident, with a larger deformation in thickness for the third mode.

**Figure 14:**
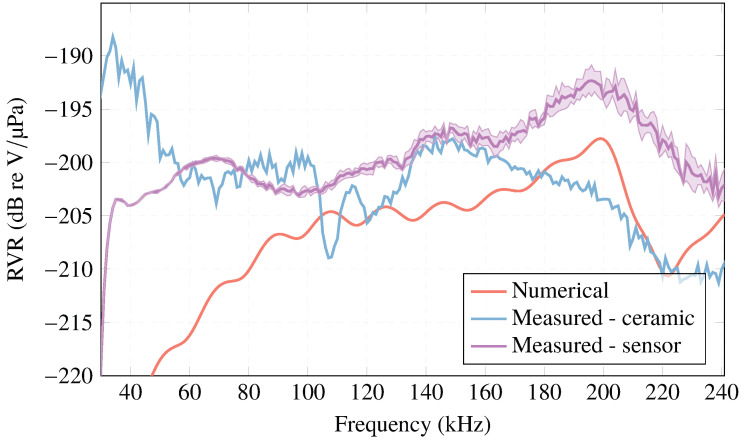
Simulated and measured RVR for ceramic and sensor in water.

Housing the ceramic improves the RVR, with a flatter response over the entire frequency sweep from 50 to 200 kHz, with a value of ∼−200 dB re V/μPa. From 174 kHz, the sensitivity is higher with a value of ∼−197 dB re V/μPa, as depicted in Figure 14. Taking into account the systematic uncertainties of the measurement, this result should be taken with caution. In these cases, the estimated systematic uncertainties due to reproducibility are 3 dB.

**Figure 15:**
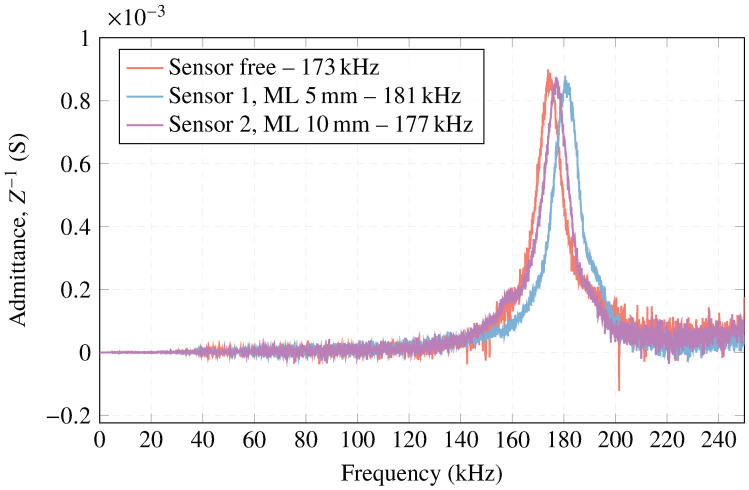
Electrical admittance measurement with the sensors attached to the matching layer compared to free sensors.

Regarding the sensors attached in different thicknesses of methacrylate as a matching layer, ML, the electrical admittance curve is presented in Figure 15 and compared with the free sensors measured in water. As can be observed, sensor 1 (ML—5 mm) and sensor 2 (ML—10 mm) produce a slight shift towards high frequencies in the admittance peak, fr=181 kHz, and 177 kHz, respectively, compared to the free sensors, 173 kHz. This increase in the resonance frequency is due to the influence that the methacrylate exerts as a matching layer of the sensors, allowing for improvement in the impedance matching of the media.

**Figure 16:**
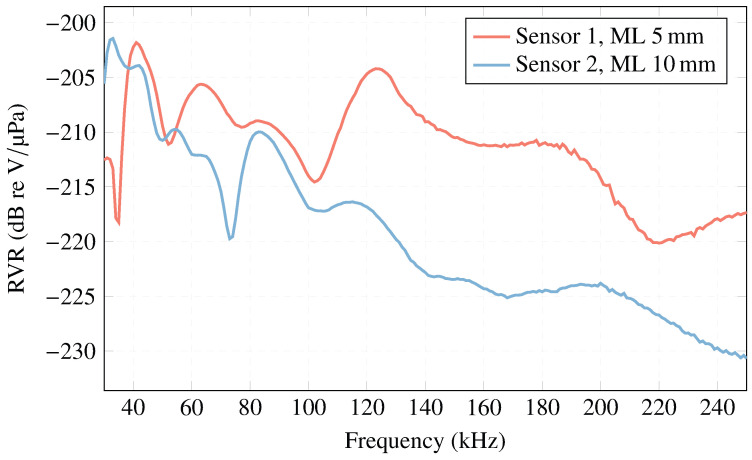
RVR curve for sensors with ML compared to sensors free in the water.

In relation to the RVR of the sensors when using a matching layer, Figure 16 shows that in sensor 1 (ML—5 mm) there is an increase of the RVR at 124 kHz (except for the lowest frequency values). This value is close to the analytical one-layer model at 134 kHz. For sensor 2 (ML—10 mm), there is a higher sensitivity at the frequency of 82 kHz, which is close to the analytical one-layer model at 67 kHz. Therefore, for these frequencies, there is a higher acoustic sensitivity.

Below, Table 2 shows the obtained results for different studies of electrical admittance.

## 5. Conclusions

While many previous studies have focused on characterizing piezoelectric acoustic transducers based on their various vibration modes and corresponding electrical impedance, the approach presented in this work goes beyond that. Specifically, we propose a novel method for characterizing a piezoelectric sensor based on its acoustic sensitivity, which is a crucial characteristic that defines the sensor’s performance. To this end, we not only design and manufacture the sensor, but also perform a comprehensive analysis of its acoustic sensitivity, which provides valuable insights for future applications of ultrasonic measurement systems.

This work proposed a comparison of three methods for designing and characterizing low-frequency piezoelectric transducers. An ultrasonic measurement system was developed to study sound propagation in the fluid medium, starting with a 1D analytical model of a ceramic vibrating in its radial mode. A second method using FEM was then applied to obtain a more realistic response of the designed sensor, with the analysis of its electrical impedance and RVR. Finally, an easy-to-manufacture and assemble sensor was constructed and characterized through experimental testing, with results compared against the first two methods. The influence of the matching layer and housing design on the sensor performance was also studied. This paper not only proposed the characterization of a sensor based on its acoustic sensitivity but also designed and manufactured such a sensor based on this important characteristic, which defines piezoelectric sensors.

The three methods proposed in this work have enabled the design and full characterization of a sensor’s role in an ultrasonic measurement system. The results obtained provide a powerful tool to analyze and optimize ultrasonic measurement systems at various levels, which can have significant practical implications.

## Figures and Tables

**Table 1:** Velocity, density, and acoustic impedance values of the elements tested in the experiment.

Element	Velocity m/s	Density kg/m${}^{3}$	Acoustic Impedance $Z_{\mathrm{ac}}$ (MRayl)
Water	1480	1000	1.48
Methacrylate	2700	119	3.21
Ceramic	4000	7800	31.2

**Table 2:** Experimental results obtained from electrical admittance for analytical, numerical, and experimental studies.

$Z^{-1}$	Analytical	Numerical	Experimental
ceramic	176 kHz	174 kHz	177 kHz±0.0011
sensor	-	-	$173\;k\mathrm{Hz}\pm 4.6942\;\cdot \;10^{-5}$
sensor (ML—5 mm)	134 kHz	-	$181\;k\mathrm{Hz}\pm 2.6456\;\cdot \;10^{-5}$
sensor (ML—10 mm)	67 kHz 202 kHz	-	$177\;k\mathrm{Hz}\pm 7.1218\;\cdot \;10^{-6}$

## Data Availability

The data presented in this study are available upon request from the corresponding author.

## Appendix A

Matrices describing the piezoelectric constants for the ceramic PIC 225.

(A1) $$S^{E}=\left[\begin{array}{cccccc} 1.59 & -0.57 & -0.74 & 0 & 0 & 0\\ -0.57 & 1.59 & -0.74 & 0 & 0 & 0\\ -0.74 & -0.74 & -2.10 & 0 & 0 & 0\\ 0 & 0 & 0 & 4.50 & 0 & 0\\ 0 & 0 & 0 & 0 & 4.50 & 0\\ 0 & 0 & 0 & 0 & 0 & 4.32 \end{array}\right]\;\cdot \;10^{-11}\left(1/Pa\right)$$

(A2) $$d=\left[\begin{array}{cccccc} 0 & 0 & 0 & 0 & 535 & 0\\ 0 & 0 & 0 & 535 & 0 & 0\\ -174 & -174 & 394 & 0 & 0 & 0 \end{array}\right]\;\cdot \;10^{-12}\left(C/N\right)$$

(A3) $$\varepsilon^{T}=\left[\begin{array}{ccc} 1649 & 0 & 0\\ 0 & 1649 & 0\\ 0 & 0 & 1750 \end{array}\right]$$

## Appendix B

Table A1 summarizes the devices employed for the characterization of measurements.

**Table A1:** Devices employed for the characterization of measurements.

Name/Brand	Serial	Description
Power amplifier	2100L	Frequency range: 10 kHz to 12 MHz, Gain: 50±1.5, Nominal output power: 100 W
Generation and acquisition, National Instruments	NI PXI-1031	The PXI platform utilized in this study was a 7-slot chassis that accommodated both DC and AC inputs. Its main purpose is to transmit a sequence of signals through channel 0, as determined by the LabVIEW software, and to receive other signals via channel 1.
NI PXI card	ExpressCard-8360	The ExpressCard-8360 is connected to a laptop computer and serves to control the PXI platform.
Motor EVA ROBOTICS	EvoDrive ST-23 FW-A201	48 V DC 3.1 A, which allows millimeter-precision steps. It is used to control the sweep in measurements requiring emitter displacements. Position resolution: $\pm 0.044{}^{\circ }$. Control resolution: ±2.5% on a 1.8 ${}^{\circ }$ motor.
Impedance analyzer Wayne Kerr Electronics	WK6500P	Frequency range: 20 Hz–5 MHz, Dissipation factor: ±0.0005, Quality factor: ±0.05%, Capacitance/Inductance/Impedance: ±0.05%
Transducer	SX60-FR	TVR: 134 dB re μPa/V @ 1 m, Capacitance: 3.6 nF±15% @ 20 ${}^{\circ }C$
Sensor Design	-	RVR: −205 dB±3 dB re V/μPa
